# Pipelle Endometrial Biopsy Versus Conventional Dilation and Curettage for the Diagnosis of Endometrial Pathology in Abnormal Uterine Bleeding

**DOI:** 10.7759/cureus.91794

**Published:** 2025-09-07

**Authors:** Swati Mishra, Richa Rathoria, Abhilasha Parihar, Shivani Singh, Sanjay Agrawal, Ekta Chaudhary, Madhulika Singh, Renu Azad, Anita Singh, Ekansh Rathoria

**Affiliations:** 1 Obstetrics and Gynecology, Hind Institute of Medical Sciences, Sitapur, IND; 2 Pathology, Hind Institute of Medical Sciences, Sitapur, IND; 3 Pediatrics, Hind Institute of Medical Sciences, Sitapur, IND

**Keywords:** abnormal, biopsy, dilatation & curettage, endometrial sampling, pipelle, uterine bleeding, uterine diseases, uterine hemorrhage

## Abstract

Introduction

Abnormal uterine bleeding (AUB) is a common issue in gynecological practice and may require evaluation depending on the patient’s age and clinical presentation. The aim of this study was to compare the diagnostic accuracy, sample adequacy, patient acceptability, and procedural outcomes of Pipelle endometrial biopsy versus dilation and curettage (D&C) in women with AUB.

Methods

A prospective observational study was conducted on 125 women aged ≥35 years presenting with AUB. All participants underwent both Pipelle biopsy and D&C sequentially. Histopathological diagnoses from both methods were compared, with D&C serving as the reference standard. Diagnostic performance metrics, sample adequacy, pain scores, procedure duration, cost, complications, and patient-reported outcomes were analyzed.

Results

Pipelle biopsy showed high diagnostic concordance with D&C (Cohen’s Kappa=0.948, p<0.001), with 97.6% agreement. Sensitivity, specificity, positive predictive value, and negative predictive value were 94.1%, 99.8%, 99.6%, and 99.5%, respectively. Sample adequacy was 97.6% for Pipelle versus 100% for D&C (p=0.247). Pipelle was significantly less painful (visual analog scale (VAS) 1.64 vs. 5.81), quicker (3.65 vs. 12.07 minutes), and more cost-effective (₹322.48 vs. ₹1387.40) than D&C (p<0.0001 for all). Pipelle had fewer complications (4% vs. 15.2%, p=0.003) and higher patient acceptability across all domains (p<0.05).

Conclusion

Pipelle endometrial biopsy is a highly accurate, safe, cost-effective, and well-tolerated alternative to conventional D&C for evaluating AUB in women aged ≥35 years. Its ease of use and outpatient feasibility support its adoption as a first-line diagnostic tool, particularly in resource-limited settings.

## Introduction

Abnormal uterine bleeding (AUB) encompasses any unexpected variation in menstrual flow, such as changes in timing, amount, or duration, that does not align with normal menstrual patterns [1]. It is among the most frequent complaints in gynecological practice and accounts for a significant proportion of outpatient visits, especially among perimenopausal women [2].

The global prevalence of AUB among reproductive-age women ranges from 3% to 30%, increasing to 35% or higher when intermenstrual and heavy menstrual bleeding (HMB) are included [3]. However, underreporting and subjective symptom interpretation contribute to variability in prevalence estimates [3]. In developing countries, AUB affects approximately 5-15% of women during their reproductive years, with a higher prevalence observed among older age groups [4].

Several studies have identified significant associations between AUB and factors such as advanced reproductive age (particularly 41-49 years), perimenopausal status, multiparity, hypertension, hypothyroidism, history of abortion, anemia, late onset of menarche (≥15 years), obesity, lower socioeconomic status, and the use of copper intrauterine devices (Cu-IUDs) [5-10].

To standardize AUB diagnosis and management, the International Federation of Gynaecology and Obstetrics (FIGO) introduced the PALM-COEIN classification system [11]. The classification system for AUB divides its causes into structural (such as polyps, adenomyosis, leiomyomas, and malignancy or hyperplasia) and non-structural categories, which include coagulopathies, ovulatory dysfunction, endometrial disorders, iatrogenic causes, and not yet classified [11]. Structural abnormalities typically require imaging or histological evaluation, while non-structural causes are diagnosed clinically or biochemically [11]. The 2018 FIGO criteria also provide standardized definitions for abnormal bleeding patterns, including frequency (e.g., amenorrhea, cycles >38 or <24 days), duration (>8 days as prolonged), regularity (cycle variation >9 days), and volume (patient-reported as light, normal, or heavy) [12]. Intermenstrual bleeding is also classified under AUB if it occurs between regular cycles. Identifying such bleeding patterns is essential for establishing an accurate diagnosis and guiding effective clinical management [12].

AUB may result from benign etiologies like hormonal imbalance or fibroids, or signal serious conditions such as endometrial hyperplasia or carcinoma [13]. Hence, histopathological assessment of the endometrium is essential in establishing a definitive diagnosis. Tissue sampling can be performed using several techniques, including D&C, hysteroscopic biopsy, and office-based methods such as Pipelle sampling [13]. Given the risk of underlying premalignant or malignant endometrial pathology, especially in women aged ≥35 years with AUB, endometrial sampling is recommended [14,15].

Dilation and curettage (D&C), developed in the 19th century by Recamier, remains a commonly performed procedure for diagnosing and treating AUB, although it is invasive and often requires anesthesia [16]. The procedure involves cervical dilation followed by scraping of the endometrial lining using curettes or cannulas. Despite its diagnostic utility, D&C poses certain risks such as uterine perforation, cervical injury, bleeding, and anesthesia-related complications [10,16].

Pipelle endometrial biopsy, introduced by Cornier in 1984, offers a less invasive outpatient alternative that does not require anesthesia or cervical dilation [17]. It utilizes a flexible suction catheter to obtain tissue. It is cost-effective, well-tolerated, and associated with fewer complications [18]. However, concerns exist regarding its ability to detect focal lesions or collect adequate samples in all cases, particularly among postmenopausal women [18,19].

Previous studies have reported high diagnostic concordance between Pipelle biopsy and D&C for common endometrial pathologies [20-23]. Pipelle endometrial biopsy may serve as a reliable and less invasive alternative to conventional diagnostic curettage [21]. When an adequate endometrial sample is obtained, a positive histopathological finding is highly specific and more effective for confirming the presence of pathology, whereas a negative result may not definitively exclude disease [21]. However, data remain limited in the Indian setting regarding comparative diagnostic accuracy, sample adequacy, patient acceptability, and procedural complications associated with these techniques.

This study primarily aimed to evaluate and compare the diagnostic accuracy of Pipelle endometrial biopsy versus D&C in women with AUB. Secondary objectives included assessing sample adequacy; evaluating the sensitivity, specificity, positive predictive value (PPV), and negative predictive value (NPV) of Pipelle biopsy using D&C as the reference standard; and comparing patient-reported pain, acceptability, complications, procedure time, cost, and anesthesia requirements between the two techniques.

## Materials and methods

This prospective observational study was conducted over 18 months (August 2023 to January 2025) in the Departments of Obstetrics and Gynaecology and Pathology at Hind Institute of Medical Sciences, Sitapur, Uttar Pradesh, India. A total of 125 women with AUB were enrolled after obtaining informed consent and ethical approval.

Women aged 35 years and above with AUB were included if endometrial evaluation was clinically indicated, they were hemodynamically stable, and eligible to undergo both Pipelle biopsy and D&C. This age threshold was chosen because AUB in women ≥35 years carries a higher risk of premalignant and malignant endometrial pathology, and histopathological assessment is recommended in this group [14,15]. The exclusion criteria comprised cases with pregnancy or bleeding related to pregnancy, as well as those with an endometrial thickness <4 mm on ultrasound, diagnosed thyroid dysfunction, tamoxifen use, active pelvic or genital tract infection, bleeding/coagulation disorders, uterine anomalies, suspected cervical cancer, recent hormonal therapy or endometrial sampling (within three months), and inability to tolerate Pipelle biopsy in an outpatient setting.

Using the standard formula for estimating sensitivity (Se) in diagnostic accuracy studies n=Z^2^.Se.(1-Se)/d^2^, where Z is 1.96 for a 95% CI, Se was 0.9688 based on a reported 96.88% sensitivity from a previous study, and the margin of error (d) was set at 0.05, the minimum number of disease-positive individuals required was approximately 47 [24,25]. Based on a disease prevalence of 50% reported in prior literature, the minimum total sample size required to achieve this was 47/0.5=94 [3]. To enhance statistical power and account for potentially inadequate or non-diagnostic samples, a total of 125 patients were enrolled using a consecutive sampling method.

All patients underwent detailed history-taking and general, abdominal, and pelvic examinations. Baseline investigations included complete blood count, random blood sugar, coagulation profile (bleeding time (BT), clotting time (CT), prothrombin time (PT), international normalized ratio (INR)), thyroid function tests (T3, T4, TSH), viral markers (HBsAg, HCV, HIV, VDRL), Pap smear, and pelvic ultrasonography to evaluate endometrial thickness. Both Pipelle biopsy and D&C were performed in a single sitting. Trained gynecologists with expertise in endometrial sampling conducted all procedures.

Step 1

Pipelle Endometrial Sampling

It was performed in the out-patient department (OPD) without anesthesia or cervical dilatation. The Pipelle device was inserted into the uterine cavity, negative pressure was created, and the device rotated to obtain an endometrial sample that was labelled as Sample A.

Step 2

*Dilation and Curettag*e

This procedure was performed under sterile conditions in a minor operating theatre under anesthesia. Cervical dilatation was done using Hegar’s dilators (up to size 7), followed by curettage with a sharp curette. The sample was labeled as Sample B.

Both specimens were preserved in 10% buffered formalin and sent to the pathology department for histopathological examination. All slides were reviewed by experienced pathology faculty who were blinded to the sampling method. Each sample was evaluated for adequacy and classified histologically as proliferative, secretory, endometrial hyperplasia (with or without atypia), polyp, atrophic endometrium, endometritis, or malignancy (adenocarcinoma or squamous cell carcinoma). Diagnostic agreement and yield of Pipelle biopsy were compared with D&C, considered the reference standard.

Pain was assessed immediately post-procedure using the visual analogue scale (VAS), ranging from 0 (no pain) to 10 (worst pain) [26]. Patients self-reported their pain scores directly on the VAS scale without investigator prompting or influence, thereby minimizing observer bias. A structured questionnaire developed by our study team was pre-validated through expert review and pilot testing on 10 patients, which included seven yes/no items assessing anxiety, discomfort, satisfaction, willingness for repeat procedure, and cost-effectiveness.

Data were recorded in Microsoft Excel 2016 (Microsoft Corp., Redmond, WA, US) and analyzed using IBM SPSS version 26 (IBM Corp., Armonk, NY, USA). Categorical variables were expressed as frequencies and percentages, and continuous variables as mean ± standard deviation (SD). Paired t-tests were used to compare mean cost, pain scores, and procedure duration. Chi-square or Fisher’s exact tests were used for categorical comparisons. Cohen’s Kappa measured diagnostic concordance between the two sampling methods. A p-value <0.05 was considered statistically significant.

## Results

The mean age of participants was 42.75±4.95 years. The average height was 157.02±4.65 cm, the mean weight was 56.57±8.41 kg, and the mean body mass index (BMI) was 22.93±3.17 kg/m². Most women were aged 35-45 years (88/125, 70.4%), belonged to lower socioeconomic strata (62/125, 49.6%), and had normal BMI (94/125, 75.2%) (Table 1). The majority were multiparous (118/125, 94.4%) with no history of prior abortion (87/125, 69.6%). HMB was the most common presenting complaint (73/125, 58.4%). On ultrasound, 55.2% (69/125) had a normal uterus, and 52.8% (66/125) had endometrial thickness between 8 mm and 11 mm. PAP smears were normal in 89.6% (112/125) of participants.

**Table 1:** Baseline demographic, clinical, and investigative characteristics of study participants (n=125) BMI: body mass index, IUCD: intrauterine contraceptive device, USG: ultrasonography, HMB: heavy menstrual bleeding

Parameters		Number	Percentage
Age group (years)	35-45	88	70.4
	46-55	37	29.6
Socioeconomic status	Upper	5	4
	Upper middle	10	8
	Lower middle	19	15.2
	Upper lower	29	23.2
	Lower	62	49.6
BMI categories (Kg/m^2^)	Underweight (<18.5)	15	12
	Normal (18.5-24.9)	94	75.2
	Overweight (25-29.9)	13	10.4
	Obese (≥30.0)	3	2.4
Parity	Primipara	7	5.6
	Multipara	118	94.4
History of previous abortion	No	87	69.6
	One	38	30.4
Contraception	Nil	81	64.8
	Barrier method	17	13.6
	Tubal ligation	7	5.6
	IUCD	20	16
Chief complaints	HMB	73	58.4
	Intermenstrual bleeding	5	4
	Irregular bleeding	26	20.8
	Post-menopausal bleeding	17	13.6
	Dysmenorrhea	4	3.2
USG findings	Normal uterus	69	55.2
	Bulky uterus	41	32.8
	Small-sized uterus	9	7.2
	Fibroid	3	2.4
	Adenomyosis	3	2.4
Endometrial thickness (mm)	4-7	25	20
	8-11	66	52.8
	12-14	24	19.2
	≥15	10	8
Pap smear findings	Normal	112	89.6
	Abnormal	13	10.4

Among the 125 patients evaluated using both Pipelle biopsy and D&C, there was an almost perfect agreement between the two methods. The Cohen’s Kappa coefficient was 0.948 (p<0.001), indicating excellent inter-method reliability (Table 2). Of the 125 cases, 122/125 (97.6%) showed concordant histopathological findings between Pipelle and D&C. Only three cases (3/125, 2.4%) had inadequate samples using the Pipelle technique. This high level of agreement suggests that Pipelle biopsy is a reliable diagnostic tool for endometrial evaluation in women with AUB, comparable to D&C. Pipelle showed high diagnostic accuracy across all categories, with an overall accuracy of 97.6% (Table 2). The overall sensitivity, specificity, PPV, and NPV were 94.1%, 99.8%, 99.6%, and 99.5%, respectively. The highest concordance was observed for hyperplasia, endometritis, and proliferative endometrium (100% across all parameters), while slightly lower sensitivity (77.8-80.0%) was noted for atrophic endometrium and polyps.

**Table 2:** Diagnostic concordance and performance metrics of Pipelle endometrial biopsy compared with D&C in women with AUB (n=125) Cohen’s Kappa coefficient = 0.948±0.016, p<0.001, indicating excellent inter-method reliability between Pipelle biopsy and D&C. D&C: dilation and curettage, SE: standard error, PPV: positive predictive value, NPV: negative predictive value, AUB: abnormal uterine bleeding

Diagnosis (D&C)	Total cases, n (%)	Matching Pipelle result	Matching cases, n (%)	Sensitivity (%)	Specificity (%)	PPV (%)	NPV (%)	Accuracy (%)
Atrophic endometrium	9 (7.2)	Atrophic	7 (5.6)	77.8	100.0	100.0	98.3	98.4
Disorganized proliferative	12 (9.6)	Disorganized proliferative	12 (9.6)	100.0	100.0	100.0	100.0	100.0
Hyperplasia (±atypia)	22 (17.6)	Hyperplasia	22 (17.6)	100.0	100.0	100.0	100.0	100.0
Polyp	5 (4.0)	Polyp	4 (3.2)	80.0	100.0	100.0	99.2	98.4
Endometritis	5 (4.0)	Endometritis	5 (4.0)	100.0	100.0	100.0	100.0	100.0
Proliferative endometrium	40 (32.0)	Proliferative	40 (32.0)	100.0	100.0	100.0	100.0	100.0
Secretory endometrium	32 (25.6)	Secretory	32 (25.6)	100.0	98.9	96.9	100.0	99.2
Overall	125 (100)	-	122 (97.6)	94.1	99.8	99.6	99.5	97.6

The mean cost of D&C (₹1387.40±86.96) was significantly higher than that of Pipelle (₹322.48±20.38) (Table 3). Similarly, the average procedure time was longer for D&C (12.07±1.47 minutes) compared to Pipelle (3.65±0.96 minutes). Pain scores, assessed using the VAS, were also significantly greater for D&C (5.81±1.14) than for Pipelle (1.64±0.89). All differences were statistically significant (p<0.0001), indicating that Pipelle biopsy is a quicker, less painful, and more cost-effective alternative to conventional D&C.

**Table 3:** Comparison of cost, time, and pain scores between Pipelle and D&C procedures (n=125) Statistical test used: paired t-test; significant p-value <0.05. D&C: dilation & curettage, SD: standard deviation, VAS: visual analogue scale

Parameters	Pipelle biopsy (mean ± SD)	D&C (mean ± SD)	t-test value	p-value
Cost (rupees)	322.48±20.38	1387.40±86.96	133.30	<0.0001
Time (minutes)	3.65±0.96	12.07±1.47	51.72	<0.0001
Pain (VAS score)	1.64±0.89	5.81±1.14	32.85	<0.0001

Sample adequacy was high in both groups, with 97.6% (122/125) for Pipelle and 100% (125/125) adequacy for D&C. The difference in adequacy was not statistically significant (p=0.247, Fisher’s exact test) (Table 4). In contrast, the use of anesthesia differed markedly between the two procedures. All D&C procedures required anesthesia (125/125, 100%), while none of the Pipelle biopsies did (0/125, 0%), a highly significant difference (p<0.0001, Fisher’s exact test). Complication rates were significantly lower with Pipelle (5/125, 4%) compared to D&C (19/125, 15.2%) (p=0.003, Chi-square test), indicating a safer profile for outpatient use. Among the 19 cases with D&C-related complications, excessive bleeding occurred in 14/19 cases (73.7%) and cervical injury in 5/19 cases (26.3%). In contrast, five cases of Pipelle-related complications were minimal cramping (2/5, 40%), minimal vaginal bleeding (2/5, 40%), and vasovagal syncope (1/5, 20%), which were mostly self-limited.

**Table 4:** Comparison of sample adequacy, anesthesia requirement, and complications between Pipelle biopsy and D&C (n=125) Data presented as n (%); significant p-value <0.05. D&C: dilation & curettage

Parameters		Pipelle, n (%)	D&C, n (%)	Statistical test	p-value
Sample adequacy	Adequate	122 (97.6)	125 (100)	Fisher’s exact test	0.247
	Inadequate	3 (2.4)	0 (0)		
Anesthesia	No	125 (100)	0 (0)	Fisher’s exact test	<0.0001
	Yes	0 (0)	125 (100)		
Complications	No	120 (96)	106 (84.8)	Chi-square test (χ²)=9.034	0.003
	Yes	5 (4)	19 (15.2)		

Table 5 presents a comparison of patient acceptability between Pipelle biopsy and D&C. Statistically significant differences were observed across most domains, including anxiety, post-procedural discomfort, willingness to repeat the procedure, and overall satisfaction, all favoring Pipelle biopsy (p<0.05).

**Table 5:** Comparison of patient acceptability and satisfaction between Pipelle biopsy and D&C (n=125) Data presented as n (%); significant p-value <0.05. D&C: dilation & curettage

Acceptability questions		Pipelle, n (%)	D&C, n (%)	Chi-square (χ²)	p-value
Was the duration of the procedure acceptable to you?	No	5 (4.0)	14 (11.2)	4.614	0.032
	Yes	120 (96.0)	111 (88.8)		
Do you feel this procedure was cost-effective?	No	5 (4.0)	17 (13.6)	7.177	0.007
	Yes	120 (96.0)	108 (86.4)		
Did you experience any anxiety before or during the procedure?	No	109 (87.2)	9 (7.2)	160.503	<0.0001
	Yes	16 (12.8)	116 (92.8)		
Did you experience any discomfort after the procedure?	No	120 (96.0)	106 (84.8)	9.034	0.003
	Yes	5 (4.0)	19 (15.2)		
Would you agree to undergo this procedure again if needed?	No	6 (4.8)	49 (39.2)	43.1	<0.0001
	Yes	119 (95.2)	76 (60.8)		
Would you recommend this procedure to other women in similar situations?	No	6 (4.8)	29 (23.2)	17.575	<0.0001
	Yes	119 (95.2)	96 (76.8)		
Were you satisfied with the overall procedure experience?	No	5 (4.0)	48 (38.4)	44.273	<0.0001
	Yes	120 (96.0)	77 (61.6)		

## Discussion

This prospective observational study compared Pipelle endometrial biopsy with D&C in women presenting with AUB, evaluating diagnostic accuracy, sample adequacy, patient acceptability, and procedural outcomes. The findings reinforce the high diagnostic reliability of Pipelle biopsy, supporting its routine use as a first-line, minimally invasive alternative to D&C.

AUB is one of the most prevalent gynecological complaints globally, especially among women in the perimenopausal age group, significantly impacting quality of life and healthcare utilization [1-4]. In line with existing literature, the majority of our participants were multiparous, aged 35-45 years, and presented with HMB, similar to findings by Sahu et al. and Bhalla et al. in Indian cohorts [7,11]. HMB remains the most reported subtype of AUB in this population, often associated with endometrial hyperplasia, fibroids, or hormonal imbalance [8,13].

Our study demonstrated excellent diagnostic agreement between Pipelle biopsy and D&C, with a Cohen’s Kappa value of 0.948 (p<0.001), and an overall diagnostic accuracy of 97.6%. These results are consistent with prior studies conducted by Gupta et al. and Khandare et al., both reporting diagnostic concordance above 90% between the two sampling methods [17,18]. A systematic review by Sakna et al. further supports these findings, highlighting the high diagnostic accuracy of Pipelle in detecting endometrial cancer, especially when adequate samples are obtained [20]. Sensitivity, specificity, PPV, and NPV of Pipelle biopsy in our study were 94.1%, 99.8%, 99.6%, and 99.5%, respectively. Notably, hyperplasia, endometritis, and proliferative endometrium were detected with 100% sensitivity and specificity, indicating that Pipelle performs particularly well in identifying diffuse endometrial pathologies.

The slightly reduced sensitivity for atrophic endometrium (77.8%) and polyps (80.0%) is consistent with prior literature suggesting that focal lesions or very scanty tissue, as seen in postmenopausal atrophy, may be missed due to the blind nature of the Pipelle device [18,19]. This underscores the importance of incorporating imaging or hysteroscopic evaluation in select patients, especially postmenopausal women or those with persistent symptoms despite a benign Pipelle result. Clark et al. have similarly emphasized the superiority of hysteroscopy in detecting focal intrauterine lesions, particularly polyps and submucous fibroids [14].

One of the most significant advantages of the Pipelle biopsy observed in this study was its minimal invasiveness. Unlike D&C, Pipelle required no anesthesia (0% vs. 100%), had a significantly shorter procedure time (3.65 vs. 12.07 minutes), and was associated with markedly lower pain scores (VAS 1.64 vs. 5.81; p<0.0001). These findings align with previous observations by Patankar and Nitnaware, who emphasized the tolerability and safety profile of Pipelle biopsy for outpatient use [13]. Atalı et al. also demonstrated improved patient comfort and reduced anxiety in less invasive procedures without general anesthesia, which resonates with the higher acceptability scores seen with Pipelle in our study [26].

In terms of complications, Pipelle demonstrated a substantially lower rate (4%) compared to D&C (15.2%; p=0.003). While most complications from D&C involved bleeding and cervical injury, those from Pipelle were self-limiting and minor. This supports the growing consensus that Pipelle biopsy offers a safer alternative in women who are not candidates for general anesthesia or in resource-limited settings where operating theatre access may be constrained. Studies by Ashraf et al. and Mathew et al. corroborate these safety outcomes, noting minimal adverse effects with Pipelle use in both pre- and postmenopausal women [19,23].

Importantly, patient-reported outcomes in this study favored Pipelle biopsy across all domains of acceptability. Women reported significantly less anxiety, less post-procedural discomfort, greater willingness to undergo repeat biopsy, and higher overall satisfaction with Pipelle compared to D&C (p<0.05 across most parameters). These findings resonate with earlier reports by Gari et al. and Ashraf et al., who found Pipelle to be more acceptable to patients due to its reduced procedural burden and cost [10,19]. Narice et al., in a meta-synthesis, also concluded that Pipelle sampling was well-tolerated in low-risk AUB cases and could reduce the need for hospital-based procedures [22].

Regarding cost, Pipelle proved to be significantly more economical, with an average expense of ₹322.48 compared to ₹1387.40 for D&C (p<0.0001). This is especially relevant in the Indian healthcare context, where out-of-pocket expenditure remains a major barrier to care. When scaled, Pipelle’s affordability could ease financial burdens for both patients and health systems. This aligns with findings from Preethi et al., who highlighted financial constraints and health system strain as major barriers to timely gynecologic care in India during and after the pandemic [8].

Sample adequacy is a critical determinant of diagnostic utility. Although D&C achieved 100% adequacy, Pipelle yielded adequate samples in 97.6% of cases, a difference that was not statistically significant (p=0.247). Prior studies have reported Pipelle sample adequacy ranging from 88% to 98%, depending on patient population and operator experience [21,22]. The high adequacy in this study may reflect standardized technique and operator training. Kanaga Lakshmi et al. also observed similar adequacy levels in perimenopausal women, supporting its effectiveness in this target population [25].

Our findings align with the goals of India’s Reproductive, Maternal, Newborn, Child and Adolescent Health (RMNCH+A) strategy, which emphasizes affordable, accessible, and high-quality reproductive health services across all levels of care [27]. In summary, this study supports the routine use of Pipelle biopsy as a first-line, office-based diagnostic tool for AUB in women aged ≥35 years. Its high accuracy, patient comfort, safety profile, and cost-effectiveness make it a practical and scalable alternative to conventional D&C, particularly in resource-limited or high-volume clinical settings.

Strengths

A key strength of this study is its prospective design with direct comparison of both techniques in the same patient, eliminating inter-subject variability. The blinding of pathologists to the sampling method further reduces bias. The study also comprehensively evaluated diagnostic performance, procedural metrics (pain, time, cost), sample adequacy, complications, and patient satisfaction, providing a holistic view of the clinical utility of Pipelle biopsy. Use of a structured and pre-validated acceptability questionnaire added rigor to patient-centered outcomes assessment.

Limitations

Some limitations should be acknowledged. This study was conducted at a single tertiary care center, which may affect generalizability. Sample adequacy and diagnostic yield with Pipelle may also vary with operator experience and pathology expertise, particularly in primary care or low-resource settings, underscoring the need for structured training programs before wider adoption. Although the sample size was adequately powered for sensitivity analysis, it may not have been sufficient to detect rare pathologies such as endometrial carcinoma. The use of D&C as the reference standard, while conventional, is also imperfect, as focal lesions or sampling errors may still occur. Excluding women with endometrial thickness <4 mm may have led to underrepresentation of atrophic or subclinical pathology. Since our study population included only women aged ≥35 years, the findings may not be directly applicable to younger women with AUB, where premalignant and malignant lesions are less common and management strategies may differ. Moreover, no malignant cases were encountered in our cohort, which limits conclusions regarding Pipelle’s diagnostic performance in detecting endometrial carcinoma. Finally, long-term follow-up of patients was not included, restricting our ability to assess clinical outcomes beyond initial diagnosis.

## Conclusions

This study confirms that Pipelle endometrial biopsy is a highly accurate, safe, cost-effective, and patient-preferred alternative to conventional D&C for evaluating AUB. Its high diagnostic concordance, minimal complications, and feasibility in outpatient settings make it especially suitable for routine use in primary and secondary care, particularly in resource-limited or high-volume centers. However, the absence of malignant cases in our cohort and the limitations of D&C as a gold standard warrant cautious interpretation. Larger multicenter studies, including malignant pathology and diverse populations, are needed to validate Pipelle’s role as a first-line diagnostic tool. Training healthcare providers in proper technique is essential to ensure sample adequacy, and integrating Pipelle into national AUB management guidelines (such as India’s RMNCH+A strategy) could support equitable, evidence-based care. In parallel, community education should be prioritized to encourage early reporting of AUB, especially in underserved areas. Overall, Pipelle biopsy represents a viable and scalable solution to strengthen gynecologic care delivery across both urban and rural health systems.
